# Development of hypertension models for lung cancer screening cohorts using clinical and thoracic aorta imaging factors

**DOI:** 10.1038/s41598-024-57396-1

**Published:** 2024-03-22

**Authors:** Jinrong Yang, Jie Yu, Yaoling Wang, Man Liao, Yingying Ji, Xiang Li, Xuechun Wang, Jun Chen, Benling Qi, Fan Yang

**Affiliations:** 1 Department of Radiology, Union Hospital, Tongji Medical College, Huazhong University of Science and Technology, Wuhan, China; 2 Department of Geriatrics, Union Hospital, Tongji Medical College, Huazhong University of Science and Technology, Wuhan, China; 3 Shanghai United Imaging Intelligence Inc., Shanghai, China; 4 Precision Healthcare Institute, GE Healthcare, Shanghai, China

**Keywords:** Health care, Medical research, Risk factors

## Abstract

This study aims to develop and validate nomogram models utilizing clinical and thoracic aorta imaging factors to assess the risk of hypertension for lung cancer screening cohorts. We included 804 patients and collected baseline clinical data, biochemical indicators, coexisting conditions, and thoracic aorta factors. Patients were randomly divided into a training set (70%) and a validation set (30%). In the training set, variance, t-test/Mann–Whitney U-test and standard least absolute shrinkage and selection operator were used to select thoracic aorta imaging features for constructing the AIScore. Multivariate logistic backward stepwise regression was utilized to analyze the influencing factors of hypertension. Five prediction models (named AIMeasure model, BasicClinical model, TotalClinical model, AIBasicClinical model, AITotalClinical model) were constructed for practical clinical use, tailored to different data scenarios. Additionally, the performance of the models was evaluated using receiver operating characteristic (ROC) curves, calibration curves and decision curve analyses (DCA). The areas under the ROC curve for the five models were 0.73, 0.77, 0.83, 0.78, 0.84 in the training set, and 0.77, 0.78, 0.81, 0.78, 0.82 in the validation set, respectively. Furthermore, the calibration curves and DCAs of both sets performed well on accuracy and clinical practicality. The nomogram models for hypertension risk prediction demonstrate good predictive capability and clinical utility. These models can serve as effective tools for assessing hypertension risk, enabling timely non-pharmacological interventions to preempt or delay the future onset of hypertension.

## Introduction

Hypertension stands as a critical risk factor of cardiovascular and cerebrovascular diseases^1–3^. It has been reported that the global prevalence of hypertension among adults is 31.1%, but its awareness, treatment and control rate remain relatively low^1,4,5^. In the context of diagnostic criteria of hypertension, systolic blood pressure ≥ 140 mmHg and/or diastolic blood pressure ≥ 90 mmHg, the prevalence rate, awareness rate, treatment rate and control rate of hypertension in Chinese population are 18.0–44.7%, 23.6–56.2%, 14.2–48.5%, 4.2–30.1%, respectively^6,7^. On November 13, 2022, China’s clinical practice guidelines for management of hypertension was released, lowering the diagnostic criteria to systolic blood pressure ≥ 130 mmHg and/or diastolic blood pressure ≥ 80mmHg^8^. This adjustment is expected to double the number of individuals with hypertension in China, reaching nearly 500 million. With the acceleration of aging process, the prevalence of hypertension will further increase. Consequently, it is imperative to institute effective public health interventions for hypertension, beginning with the identification and screening of individuals at high risk.

Several hypertension prediction models have emerged. For instance, the Framingham Heart Study developed a short-term hypertension prediction model, encompassing conventional factors such as age, gender, baseline blood pressure, body mass index (BMI), parental history of hypertension, and smoking^9^. Nonetheless, its applicability might be limited to Caucasian non-diabetic patients^10^, restricting its extension to non-white races or diabetics^11^. Asian nations, including China, Japan and South Korea, have also established regional hypertension prediction models that incorporate both conventional and local risk factors^12–21^. However, these models only contain part of the influencing factors.

With the advancing comprehension of hypertension's pathophysiology, there is growing emphasis on early detection of hypertension-induced vascular structural and functional changes. Current indicators for early detection including dilatation, stiffness, and compliance, reflecting variations in vascular lumen and wall, offering insights into wall pressure and vascular function^22–24^. Predominantly, manual measurement techniques involve ultrasound, gated chest CT, and MRI, with a primary focus on the diameter of the ascending aorta^25–28^. Studies have demonstrated an interrelationship between dilatation of the ascending aorta and the risk of cardiovascular diseases, notably hypertension. Hypertension is an independent risk factor for vascular dilatation, which in turn is a predictor of cardiovascular diseases^29,30^. Given the prevalence of chest CT scans in lung cancer screenings, its images carry valuable potential information about the thoracic aorta^31^. Deep learning-powered artificial intelligence has emerged as an innovative approach for quantitative analysis of CT images. In non-contrast chest CT scans, AI enables automated quantitative analysis of the thoracic aorta.

The objective of this study is to develop and validate hypertension prediction models utilizing clinical risk factors and thoracic aorta characteristics, subsequently assessing the models' effectiveness.

## Methods

### Patients

This study was approved by the Ethics Committee of Wuhan Union Hospital ([2021]0853) in accordance with the Declaration of Helsinki. The research is retrospective and the data are anonymous, thus informed consent was waived by Wuhan Union Hospital Ethics committee.

**Figure 1:**
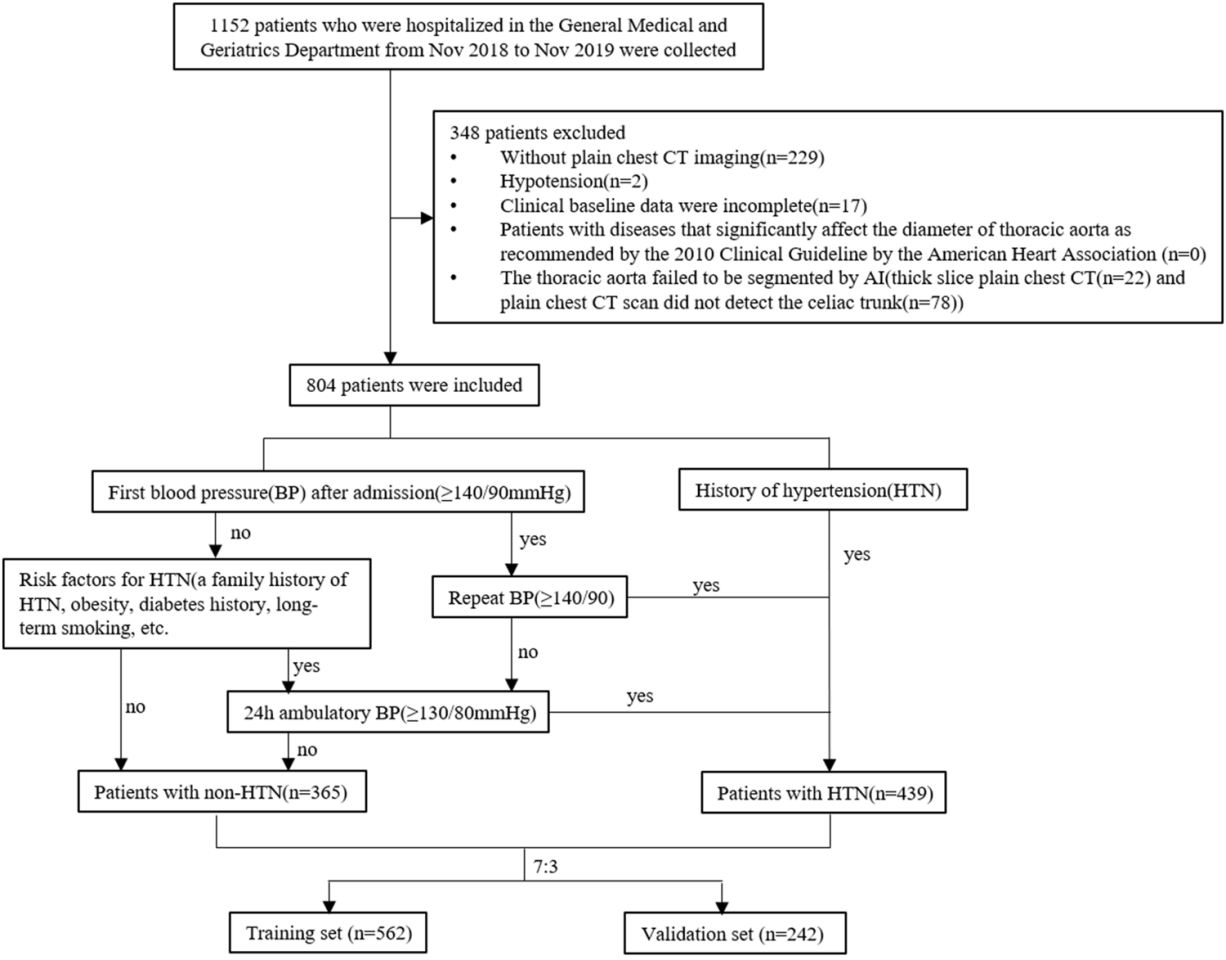
Flowchart illustrating the inclusion criteria and grouping of patients.

From November 2018 to November 2019, patients who were hospitalized in the General Medical and Geriatrics Department were included. After excluding 348 patients who did not meet the criteria (see Fig. 1 for details), 804 patients were eventually included in the study, of whom 439 had hypertension and 365 were without hypertension. And blood pressure measurement and diagnosis of hypertension are carried out strictly by professional doctors according to 2018 ESC/ ESH Guidelines for the management of arterial hypertension^32^. The whole patients were then completely randomly divided into the training cohort and internal validation cohort by a ratio of seven to three. The flowchart presented the detailed procedures of the inclusion and exclusion criteria, blood pressure monitoring and diagnosis as well as grouping.

Baseline clinical data, including age, sex, height, weight, personal history, serum biochemical markers and other concomitant diseases were acquired from the electronic medical records system.

### CT image acquisition

All the included patients underwent non-contrast enhanced chest CT (NCCT) examinations during hospitalization. All chest CT images were acquired from thoracic inlet to the diaphragm with patients in a supine position. Scans were performed using one of the following three CT scanners: SIEMENS- Definition, Germany; GE-Discovery CT750HD, USA; TOSHIBA-Aquilion ONE, Janpan. And applied scanning parameters: tube voltage 120 kV, self-regulating tube current by automatic exposure control system. The reconstruction slice thickness of the chest CT images was 1–2 mm, and the reconstruction interval was 1–2 mm.

### Thoracic aorta segmentation and features extraction

**Figure 2:**
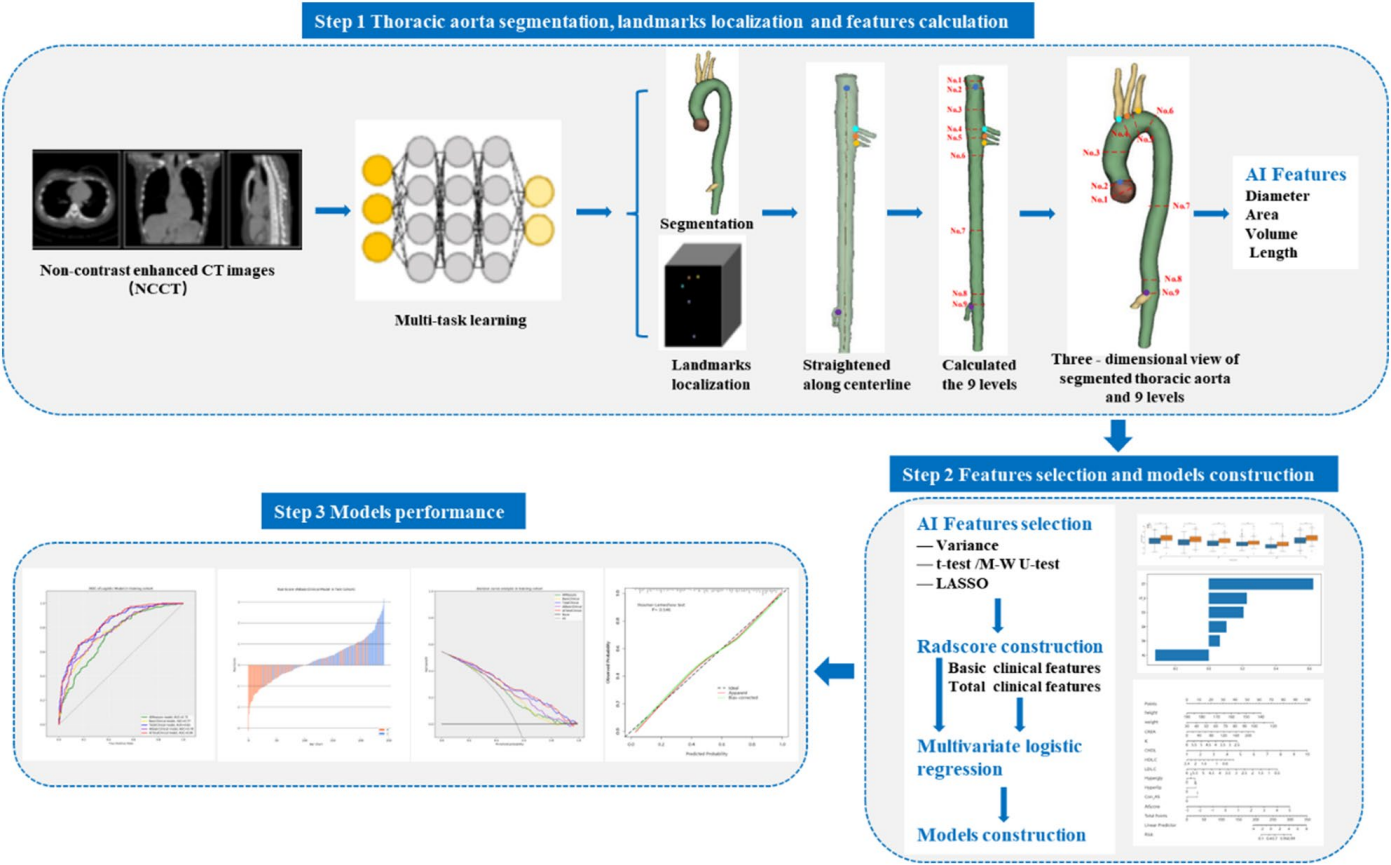
The workflow of the research.

In this study, a multi-task learning framework (provided by Shanghai United Imaging Intelligence Co. Ltd., Shanghai, China) was used to automatically measure nine key positions of the thoracic aorta recommended by AHA guidelines^33^ on chest CT images (Fig. 2, Supplementary Fig. S1). The framework is an automatic post-processing tool for thoracic aorta utilized deep learning methods, which has been applied and validated on challenging NCCT images.

The automatic quantification of thoracic aorta mainly consists of three tasks: aortic segmentation, aortic anatomical marker detection and aortic measurement. Using two parallel subnets, the framework can simultaneously accomplish two tasks of aortic segmentation and thoracic aortic anatomical landmark localization. Specifically, the segmentation subnetwork was intended to delineate the thoracic aortic boundary, and the detection subnetwork was intended to detected five key anatomical landmarks, including the aortic sinus, brachiocephalic artery, left common carotid artery, left subclavian artery, and celiac trunk. Based on the segmented aortic mask and the five detected anatomical landmarks, the nine key landmarks recommended by the AHA guidelines can be inferred. Thus, we can calculate the diameters and cross-sectional areas of the nine landmarks as well as the total and segmental volume and length. More details and performance tests for each section can be found in the reference^34^.

### Feature selection and development, validation of signature

A three-step procedure was performed for dimensionality reduction for thoracic aorta imaging factors. Firstly, thoracic aorta imaging factors with variance > 1.0 were selected. Secondly, Analysis of Variance was applied to choose the statistical influence feature for hypertension. Finally, the thoracic aorta imaging factors met the criteria of variance > 1.0 and being significantly different between non-HTN group and HTN group were enrolled into the least absolute shrinkage and selection operation (LASSO) regression method to select the most related features with non-zero coefficients from the training cohort.

After feature selection, the AIScore was computed for each patient through the LASSO regression with a combination of selected features weighted by their respective coefficients. Both feature selection and AIScore development were performed in the training cohort. And it was evaluated in the internal validation set.

### Construction of different models

In order to meet the needs of different clinical application scenarios, we established five models with different features: AIMeasure model, BasicClinical model, TotalClinical model, AIBasicClinical model and AITotalClinical model.

AIMeasure model was constructed as the sum of weights of the selected thoracic aorta imaging factors using logistic regression. The clinical factors models were comprised of BasicClinical model and TotalClinical model, which were built using the directly available basic clinical data (including sex, age, height, weight, BMI, smoking history, drinking history, etc.) and the total clinical data (including the above basic clinical data, serum biomarkers and concomitant diseases, etc.), respectively. Concretely, we first applied univariate analysis to compare the differences of clinical factors between the two groups. Then the variables with significant statistical differences were inputted into the multivariate logistic backward stepwise regression to build the clinical factors models. Further, we established the AIBasicClinical model and AITotalClinical model by combining valuable clinical factors and AI-score using the multivariate logistic backward stepwise regression method.

### Assessment of the performance of different models

The predictive performance of the five models for identifying hypertension was evaluated using receiver operating characteristic (ROC) curve in both the training set and validation set. At the same time, Delong test was used to compare the differences of AUC values among different models. The agreement between the predicted and actual probabilities of the models was appraised with a calibration curve. To assess the clinical applicability of the five models, a decision curve analysis (DCA) was carried out by calculating the net benefits.

### Statistical analysis

The statistical analysis was performed using R software (version 4.0.3, https://www.r-project.org). And *p* < 0.05 was considered statistically significant.

The Shapiro-Wilks method was used for normal distribution test, and the Levene method was used for homogeneity of variance test. The independent-samples t-test (for normal distribution continuous variables), Mann–Whitney U-test (for non-normal distribution continuous variables) and chi-square test (for categorical variables) were used to compare the differences of clinical factors between two groups. Normally distributed variables were presented as mean ± standard deviation, non-normally distributed variables were expressed as median (25th and 75th percentile), and categorical variables were expressed as count (percentage). Difference of thoracic aorta imaging factors was compared using ANOVA analysis.

The “glmnet” package was used for standard LASSO regression. The “rms” package was used to perform the multivariate binary logistic regression and develop nomogram. The “pROC” package was applied to plot ROC curves and Delong test was used to estimate the differences of AUC values among different models. And calibration curves were plotted using the “rms” package, while the DCA was carried out using the “rmda” package.

## Results

### Characteristics of patients

A total of 804 patients who met the inclusion criteria were finally enrolled in this study (median age was 52 years, male accounted for 30.6%), including 439 patients with hypertension (54.6%) (Fig. 1, Table 1). Detailed clinical features of the patients are presented in Table 1. None of the clinical features were statistically different between the training set and validation set.

**Table 1:** Comparison of clinical features between the HTN group and non-HTN group, as well as between the training set and validation set.

Features	Non-HTN (n = 365)	HTN (n = 439)	Statistic value	*P* value	Training set (n = 562)	Validation set (n = 242)	Statistic value	*P* value
Age	52.00 (45.00, 59.30)	52.00 (51.00, 70.00)	− 8.498	< 0.001	55.00 (49.00, 66.00)	55.00 (47.95, 62.00)	− 1.291	0.197
Sex (male%)	150 (41.10%)	96 (21.87%)	34.699	< 0.001	170 (30.25%)	76 (31.40%)	0.106	0.744
Height	167.00 (160.00, 172.00)	167.00 (164.20, 174.00)	− 3.003	0.003	168.00 (163.00, 173.00)	168.00 (162.00, 172.00)	− 0.596	0.551
Weight	64.70 (56.40, 73.00)	64.70 (64.62, 79.00)	− 8.168	< 0.001	69.80 (60.20, 76.51)	69.80 (60.58, 77.71)	0.25	0.802
BMI	23.00 (21.37, 25.30)	23.00 (23.42, 27.10)	− 9.452	< 0.001	24.25 (22.30, 26.50)	24.25 (22.30, 26.60)	0.289	0.773
GLU	4.78 (4.45, 5.20)	4.78 (4.73, 5.80)	− 7.265	< 0.001	4.96 (4.59, 5.60)	4.96 (4.64, 5.50)	− 0.063	0.95
CHOL	4.52 (3.94, 5.26)	4.52 (3.51, 5.08)	3.723	< 0.001	4.51 (3.67, 5.15)	4.51 (3.79, 5.19)	1.244	0.214
TG	1.12 (0.87, 1.57)	1.12 (1.10, 2.56)	− 8.062	< 0.001	1.36 (0.98, 2.08)	1.36 (0.99, 2.17)	0.074	0.941
HDL.C	1.26 (1.07, 1.54)	1.26 (0.92, 1.26)	8.414	< 0.001	1.16 (0.96, 1.38)	1.16 (0.99, 1.42)	0.972	0.331
LDL.C	2.78 (2.29, 3.33)	2.78 (1.86, 3.16)	4.3	< 0.001	2.75 (2.02, 3.23)	2.75 (2.15, 3.32)	1.192	0.233
ALT	20.00 (14.00, 29.00)	20.00 (16.00, 32.00)	− 3.202	0.001	22.00 (15.00, 31.00)	22.00 (15.00, 31.00)	0.287	0.774
AST	20.00 (17.00, 25.00)	20.00 (18.00, 26.00)	− 1.691	0.091	21.00 (17.95, 25.00)	21.00 (18.00, 25.00)	0.057	0.955
BUN	4.94 (4.18, 5.70)	4.94 (4.49, 6.35)	− 4.197	< 0.001	5.12 (4.29, 6.08)	5.12 (4.35, 5.85)	− 0.867	0.386
CREA	68.30 (58.50, 77.26)	68.30 (65.88, 85.28)	− 7.187	< 0.001	71.40 (62.10, 82.20)	71.40 (62.09, 80.53)	− 0.713	0.476
Na	140.60 (139.37, 141.60)	140.60 (139.50, 141.88)	− 0.977	0.328	140.60 (139.40, 141.80)	140.60 (139.50, 141.60)	− 0.209	0.835
K	3.97 (3.81, 4.13)	3.97 (3.71, 4.11)	3.232	0.001	3.96 (3.75, 4.12)	3.96 (3.80, 4.12)	1.161	0.246
Ca	2.23 (2.16, 2.31)	2.23 (2.15, 2.31)	0.597	0.55	2.22 (2.15, 2.32)	2.22 (2.15, 2.30)	− 0.973	0.331
Mg	0.87 (0.83, 0.92)	0.87 (0.82, 0.93)	0.103	0.918	0.88 (0.82, 0.92)	0.88 (0.83, 0.92)	0.833	0.405
CL	102.40 (101.00, 103.60)	102.40 (100.50, 103.78)	1.51	0.131	102.20 (100.80, 103.70)	102.20 (100.70, 103.50)	− 0.386	0.699
PHOS	1.01 (0.93, 1.12)	1.01 (0.90, 1.10)	1.433	0.152	1.02 (0.91, 1.11)	1.02 (0.91, 1.11)	0.45	0.653
CO2	25.60 (24.20, 27.03)	25.60 (23.72, 26.90)	1.337	0.181	25.40 (24.10, 27.00)	25.40 (23.80, 26.81)	− 1.533	0.125
Hypergly	77 (21.10%)	210 (47.83%)	62.96	< 0.001	203 (36.12%)	84 (34.71%)	5.777	0.056
Hyperuri	49 (13.42%)	126 (28.70%)	27.315	< 0.001	131 (23.31%)	44 (18.18%)	2.612	0.106
Hyperlip	135 (36.99%)	223 (50.80%)	15.39	< 0.001	248 (44.13%)	110 (45.45%)	0.12	0.729
Peri_AS	235 (64.38%)	391 (89.07%)	70.436	< 0.001	442 (78.65%)	184 (76.03%)	0.671	0.413
Con_AS	92 (25.21%)	216 (49.20%)	48.563	< 0.001	217 (38.61%)	91 (37.60%)	0.073	0.787
CHD	34 (9.32%)	108 (24.60%)	32.024	< 0.001	107 (19.04%)	35 (14.46%)	2.436	0.119
Cere_AS	47 (12.88%)	126 (28.70%)	29.554	< 0.001	125 (22.24%)	48 (19.83%)	0.58	0.446
Lacunar infarction	127 (34.79%)	244 (55.58%)	34.65	< 0.001	271 (48.22%)	100 (41.32%)	3.239	0.072
OP	56 (15.34%)	74 (16.86%)	0.337	0.562	92 (16.37%)	38 (15.70%)	0.056	0.814
CKD	360 (98.63%)	401 (91.34%)	20.901	< 0.001	533 (94.84%)	228 (94.21%)	0.131	0.718
COPD	5 (1.37%)	20 (4.56%)	6.714	0.01	19 (3.38%)	6 (2.48%)	0.456	0.499
Fatty liver	125 (34.25%)	241 (54.90%)	34.272	< 0.001	249 (44.31%)	117 (48.35%)	1.114	0.291
Smoking	104 (28.49%)	165 (37.59%)	7.4	0.007	188 (33.45%)	81 (33.47%)	0	0.996
Drinking	88 (24.11%)	143 (32.57%)	6.973	0.008	162 (28.83%)	69 (28.51%)	0.008	0.928

*Non-HTN* non-Hypertension; *HTN* Hypertension; *BMI* body mass index; *GLU* fasting blood glucose; *CHOL* Cholesterol; *TG* Triglyceride; *HDL.C* high-density lipoprotein cholesterol; *LDL.C* low-density lipoprotein cholesterol; *ALT* alanine aminotransferase; *AST* aspartate aminotransferase; *BUN* blood urea nitrogen; *CREA* creatinine; *Na* serum sodium; *K* serum potassium; *Ca* serum calcium; *Mg* serum magnesium; *CL* serum chlorine; *PHOS* serum phosphorus; *CO2* carbon dioxide; *Hypergly* Hyperglycemia; *Hyperuri* Hyperuricemia; *Hyperlip* Hyperlipidemia; *Peri_AS* peripheral arteriosclerosis; *Con_AS* coronary arteriosclerosis; *CHD* coronary heart disease; *Cere_AS* cerebral arteriosclerosis; *OP* osteoporosis; *CKD* chronic kidney disease; *COPD* chronic obstructive pulmonary disease.

In terms of basic clinical features, all the features including age, sex, height, weight, BMI, history of smoking and drinking were significantly different between HTN and non-HTN groups. And high blood pressure was associated with an older age, a greater BMI as well as a history of smoking and drinking. In addition, high blood pressure seems to favor women.

Concerning serum biochemical markers, HTN patients tend to have higher blood glucose and higher blood lipids. Besides, alanine aminotransferase (ALT), blood urea nitrogen (BUN), creatinine (CREA) and serum potassium (K) were also statistically different in HTN and non-HTN groups.

When it came to comorbidities, the number of patients who had hypertension with comorbidities was significantly higher than the non-HTN group. Hyperglycemia, hyperlipidemia, hyperuricemia, peripheral atherosclerosis, coronary atherosclerosis, cerebral atherosclerosis, lacunar cerebral infarction, fatty liver and other diseases tend to occur more in hypertensive patients.

### Features extraction, selection and establishment of signature

**Figure 3:**
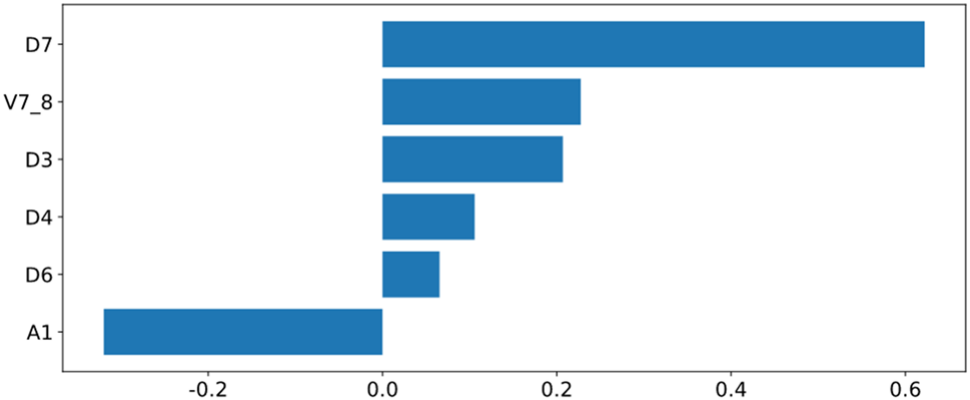
The weights of selected thoracic aorta features measured by AI. The numerical value represents a specific level of the thoracic aorta; D, A, V, and L denote the diameter, area, volume, and length of the thoracic aorta at a specific level or two adjacent levels.

In total, 43 features including the diameter and area of 9 levels of thoracic aorta, the volume and length at two adjacent levels, the volume and length of ascending aorta, aortic arch, descending aorta, and the total volume and length were obtained by AI on non-contrast enhanced chest CT (see Supplementary Table S1 and Fig. S1 for details). After excluding features with variance > 1, the remaining irrelevant redundant features were continued to be excluded by one-way ANOVA and LASSO regression. In the end, the six most relevant features were selected. The selected features and their corresponding coefficients are shown in Fig. 3. Then the AIScore is established by using the selected features and their coefficients. The AIScore showed a statistically significant difference between the HTN and non-HTN groups (Supplementary Fig. S2).

### Development of nomogram

In order to adapt to different clinical application scenarios, we established five different models based on different clinical and imaging features.

In clinical model, the method of backward stepwise logistic regression showed that age, height, weight, serum biomarkers (including cholesterol (CHOL), high-density lipoprotein cholesterol (HDL.C), low density lipoprotein cholesterol (LDL.C), creatinine (CREA), serum potassium (K) and accompanied diseases (including hyperlipidemia (hyperlip), peripheral artery atherosclerosis (peri_AS) and coronary atherosclerosis (con_AS) is the risk predictor of hypertension. Since age, height and weight can be obtained directly, we used these three features to build the BasicClinical model (Supplementary Fig. S3A), and combined them and serum markers as well as concomitant diseases to establish the TotalClinical model (Supplementary Fig. S3B). Subsequently, we used multiple logistic regression to construct an AIMeasure model utilizing the six features of thoracic aorta screened previously. Finally, both clinical features and AIScore were included in multiple stepwise backward logistic regression to construct two mixed models called AIBasicClinical Model (Supplementary Fig. S3C) and AITotalClinical model (Supplementary Fig. S3D).

The nomograms of the five models are shown in Supplementary Fig. S3, and the selected valuable features and coefficients included in the clinical and mixed models are listed in Table 2.

**Table 2:** The coefficients of features included in the two clinical models and two mixed models.

	Coef	95%CI	SE	z	P >|z|		Coef	95%CI	SE	z	P >|z|
BasicClinical Model						AIBasicClinical Model					
Age	0.013	0.010–0.015	0.001	9.227	< 0.001	Age	0.006	0.002–0.010	0.002	3.095	0.002
Height	− 0.012	− 0.018–− 0.005	0.003	− 3.572	< 0.001	Height	− 0.014	− 0.020–− 0.008	0.003	− 4.279	< 0.001
Weight	0.019	0.0151–0.023	0.002	9.389	< 0.001	Weight	0.017	0.013–0.021	0.002	8.077	< 0.001
						AIScore	0.122	0.068–0.177	0.028	4.431	< 0.001
TotalClinical Model						AITotalClinical Model					
Age	0.008	0.005–0.011	0.002	4.988	< 0.001	Height	− 0.015	− 0.021–− 0.009	0.003	− 4.684	< 0.001
Height	− 0.013	− 0.019–− 0.006	0.003	− 3.838	< 0.001	Weight	0.010	0.006–0.014	0.002	4.658	< 0.001
Weight	0.013	0.009–0.018	0.002	6.118	< 0.001	CHOL	0.095	0.027–0.162	0.035	2.732	0.006
CHOL	0.093	0.024–0.162	0.035	2.655	0.008	HDL.C	− 0.215	− 0.345–− 0.085	0.066	− 3.239	0.001
HDL.C	− 0.217	− 0.349–− 0.086	0.067	− 3.238	0.001	LDL.C	− 0.119	− 0.197–− 0.040	0.040	− 2.969	0.003
LDL.C	− 0.125	− 0.204–− 0.046	0.040	− 3.093	0.002	CREA	0.003	0.001–0.005	0.001	2.595	0.010
CREA	0.003	0.001–0.005	0.001	2.333	0.020	K	− 0.149	− 0.254–− 0.043	0.054	− 2.765	0.006
K	− 0.175	− 0.281–− 0.069	0.054	− 3.223	0.001	Hypergly	0.050	0.006–0.094	0.023	2.222	0.026
Hyperlip	0.111	0.024–0.198	0.044	2.508	0.012	Hyperlip	0.110	0.027–0.192	0.042	2.591	0.010
Peri_AS	0.105	0.005–0.205	0.051	2.050	0.040	Peri_AS	0.119	0.042–0.196	0.039	3.042	0.002
Con_AS	0.103	0.023–0.184	0.041	2.506	0.012	AIScore	0.138	0.099–0.177	0.020	6.929	< 0.001

The abbreviations align with those in Table 1.

### Evaluation and comparison of performance of different models

**Figure 4:**
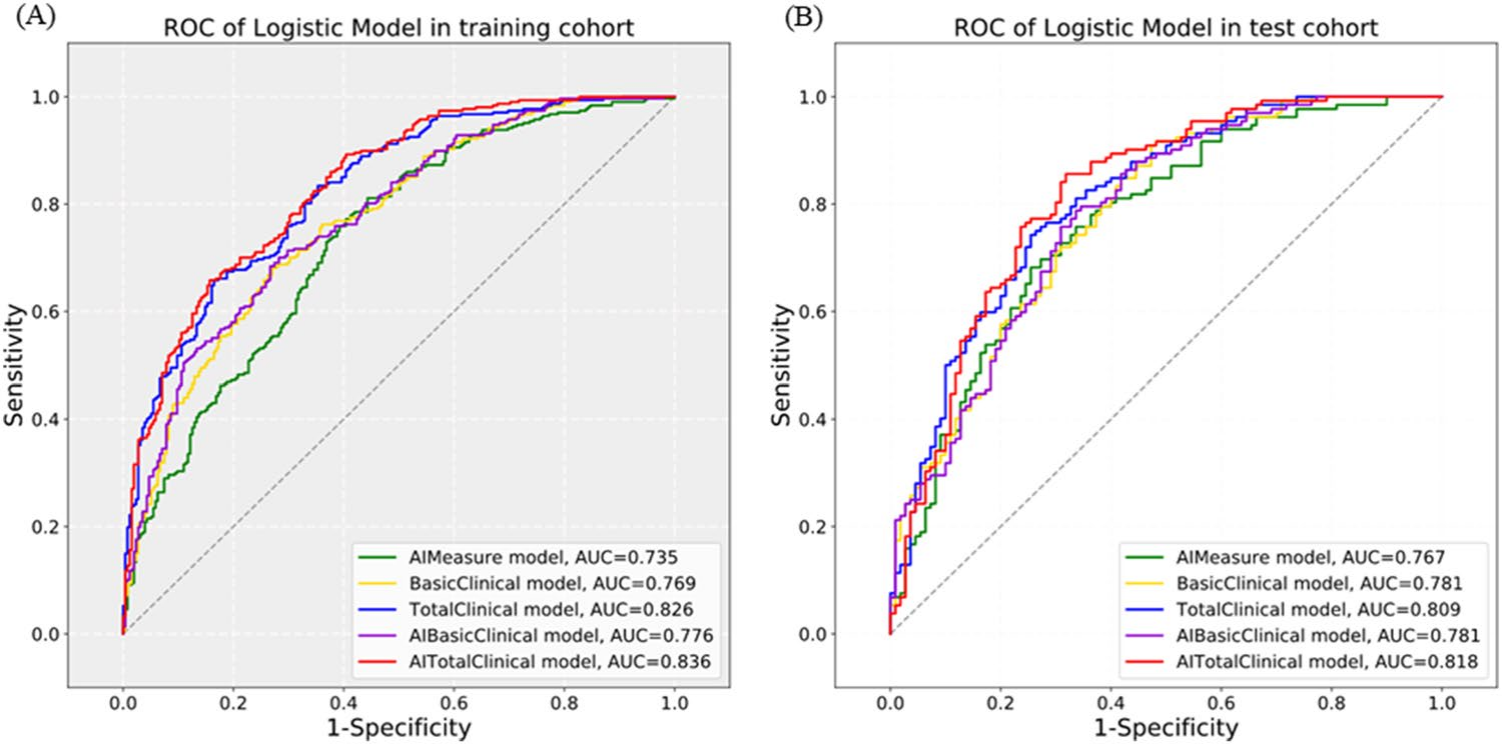
The ROC curves for the five models in the training set (**A**) and validation set (**B**).

The ROC curves of the five models in the training and validation sets were shown in Fig. 4, and the diagnostic performance was summarized in Table 3. The results presented that all the five models had good diagnostic performance for hypertension in both the training set (AUC 0.735–0.836, sensitivity 65.5–73.3%, specificity 66.7–72.5%) and the validation set (AUC 0.767–0.818, sensitivity 63.6–68.2%, specificity 70.9–77.3%). Subsequently, we compared the AUC among the five models (Table 4). In the training set, the AUC values of TotalClinical Model and AITotalClinical model were statistically different from other models (*P* < 0.001), while in the validation set, there was no significant difference in AUC among the five models (*P* > 0.05).

**Table 3:** Diagnostic performance of the five models in training set and validation set.

Models	AUC (95%CI)	Sensitivity (95%CI)	Specificity (95%CI)	Precision (95%CI)	Accuracy (95%CI)
The training set					
AIMeasure model	0.735 (0.694–0.776)	0.655 (0.652–0.658)	0.667 (0.663–0.670)	0.703 (0.700–0.706)	0.660 (0.658–0.662)
BasicClinical model	0.769 (0.731–0.808)	0.697 (0.694–0.700)	0.698 (0.695–0.702)	0.735 (0.732–0.738)	0.698 (0.696–0.699)
TotalClinical model	0.826 (0.793–0.860)	0.720 (0.717–0.723)	0.714 (0.710–0.717)	0.752 (0.749–0.755)	0.717 (0.716–0.719)
AIBasicClinical model	0.776 (0.738–0.814)	0.700 (0.697–0.703)	0.706 (0.702–0.709)	0.741 (0.738–0.744)	0.703 (0.701–0.704)
AITotalClinical model	0.836 (0.804–0.869)	0.733 (0.730–0.736)	0.725 (0.722–0.729)	0.763 (0.760–0.766)	0.730 (0.728–0.731)
The validation set					
AIMeasure model	0.767 (0.707–0.827)	0.636 (0.629–0.644)	0.755 (0.747–0.762)	0.757 (0.749–0.764)	0.690 (0.686–0.694)
BasicClinical model	0.781 (0.723–0.839)	0.652 (0.644–0.659)	0.709 (0.701–0.717)	0.729 (0.721–0.736)	0.678 (0.674–0.681)
TotalClinical model	0.809 (0.754–0.863)	0.682 (0.675–0.689)	0.764 (0.756–0.771)	0.776 (0.760–0.783)	0.719 (0.715–0.723)
AIBasicClinical model	0.781 (0.723–0.839)	0.674 (0.667–0.681)	0.718 (0.710–0.726)	0.742 (0.735–0.749)	0.694 (0.690–0.698)
AITotalClinical model	0.818 (0.763–0.872)	0.674 (0.667–0.681)	0.773 (0.765–0.780)	0.781 (0.774–0.788)	0.719 (0.715–0.723)

*AUC* area under the ROC curve.

**Table 4:** Comparison matrix of AUC for the five models in training set and validation set.

Models	AIMeasure model	BasicClinical model	TotalClinical model	AIBasicClinical model	AITotalClinical model
AIMeasure model	**1.000**	*0.608*	*0.172*	*0.616*	*0.054*
BasicClinical model	0.066	**1.000**	*0.176*	*0.968*	*0.114*
TotalClinical model	< 0.001	< 0.001	**1.000**	*0.156*	*0.432*
AIBasicClinical model	0.031	0.047	< 0.001	**1.000**	*0.101*
AITotalClinical model	< 0.001	< 0.001	0.207	< 0.001	**1.000**

In the bottom left corner, the chart illustrates the differences in AUC among the five models in the training set, while the italics in top right corner depicts the differences in AUC among the five models in the validation set. The bold in diagonal line represents self-comparisons of the five models. And Delong test was applied for all comparisons.

**Figure 5:**
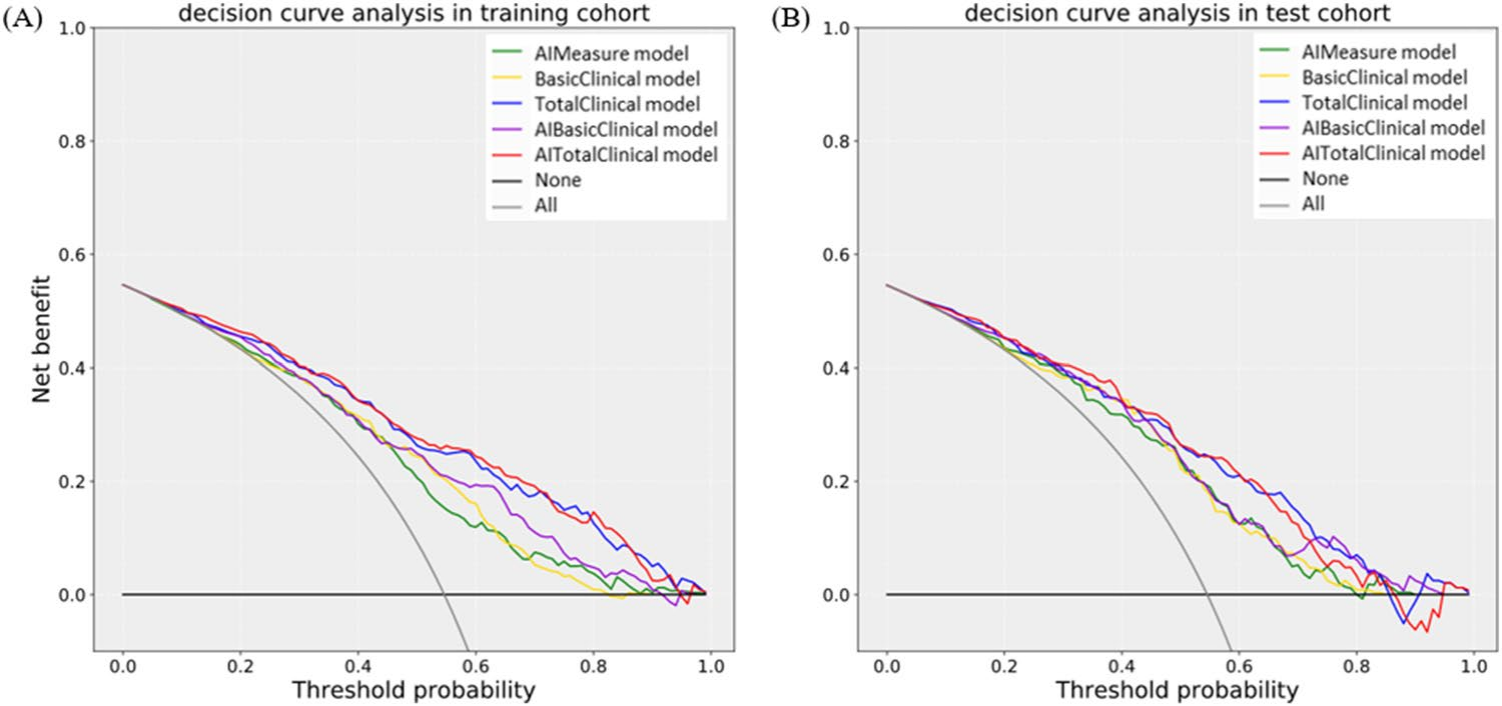
Decision curve analysis (DCA) curves for the five models in training set (**A**) and validation set (**B**).

**Figure 6:**
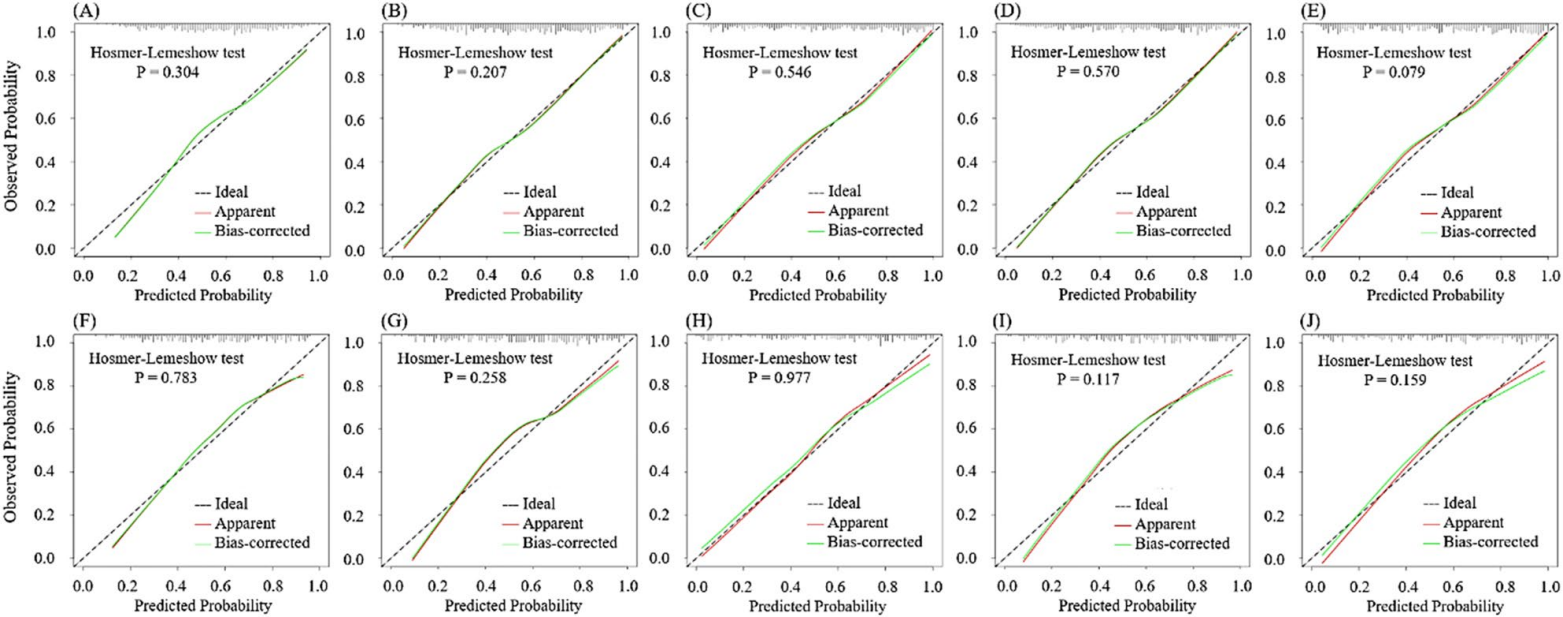
Calibration curves for the five models in training set (**A**–**E**) and validation set (**F**–**J**). From left to right, the figures depict the AIMeasure model, BasicClinical model, TotalClinical model, AIBasicClinical model and AITotalClinical model, arranged with the training set at the top and the test set at the bottom. The dashed line represents the ideal prediction line. The red line illustrates the predictive efficacy of the nomogram in hypertension prediction. The green line indicates bias correction in the model.

Calibration curve and Hosmer–Lemeshow test showed that the five models presented good calibration ability in both the training set (*P* = 0.079–0.570) and the validation set (*P* = 0.117–0.977) (Fig. 6). The decision curve analysis of the five models was shown in Fig. 5, which showed that the five models could bring net benefits to patients in most reasonable threshold probability ranges (Fig. 6).

## Discussion

This study develops five distinct hypertension risk prediction models customized for various clinical scenarios. The BasicClinical model incorporates readily available factors like age, height, and weight, making it applicable to a broad audience. Expanding on the BasicClinical model, the TotalClinical model integrates additional parameters, including blood glucose, blood lipids, electrolyte levels, comorbidities, and other clinician-assessed factors—typically gathered during physical examinations and prior medical visits. The AIMeasure model encompasses dimensions such as the diameter, cross-sectional area, volume and length of thoracic aorta. These metrics can be efficiently measured by AI using standard non-enhanced chest CT scans. Consequently, patients can simultaneously estimate hypertension risk during lung cancer screenings or pulmonary nodule follow-ups, amplifying the value of chest CT assessments. The AIBasicClinical model and the AITotalClinical model include the AIScore and the aforementioned clinical risk factors. Our hypertension risk prediction model exhibits robust calibration and substantial clinical utility. Physicians can anticipate hypertension risk based on established factors, allowing for effective preventative measures or treatment strategies.

Notably, in our initial stages of data processing, we also diligently applied the k-fold method, generating thousands of models and meticulously evaluating each one. We observed a considerable degree of stability in these results, prompting us to adopt the 7:3 random division method for the final analysis presented in the paper. The robustness of various sampling outcomes is satisfactory, and we attribute this to our ample sample size, particularly within the specific target population of individuals undergoing lung cancer screening.

Traditional risk factors for high blood pressure comprise age, BMI, smoking and others. Age serves as an independent predictor of hypertension due to diminished vascular elasticity, sluggish blood flow, and heightened blood viscosity^35^. Studies have yielded inconclusive findings regarding the association between hypertension and gender^9,36^. Within this study, women exhibited a slightly elevated hypertension incidence compared to men, potentially influenced by a greater female representation in the sample. A European study indicated a notably lower incidence of cardiovascular disease in women compared to men up to age 45, with no substantial variance in prevalence by age 60, potentially attributed to estrogen's protective impact on blood vessels^37^. Studies have shown that obesity, especially abdominal obesity, independently heightens the risk of hypertension^9,38,39^. As obesity rates surge, the incidence of high blood pressure escalates. Furthermore, smoking and alcohol consumption also contribute to an elevated hypertension risk^9,10,40,41^.

Biochemical markers obtained from routine medical check-ups and visits for other conditions provide valuable insights. This study focused on parameters such as blood glucose, lipid profiles, electrolytes and comorbidities. After rigorous variable screening, the model integrated cholesterol, high-density lipoprotein, low-density lipoprotein, blood potassium, creatinine, hyperlipidemia, and arteriosclerosis. Anomalies in glucose metabolism heighten hypertension risk by damaging blood vessels^14^. Concurrently, hyperlipidemia significantly amplifies hypertension risk, and the two often co-exist to accelerate arteriosclerosis^42^. The mechanism may involve the rise and fluctuation of blood pressure increasing stress on the vascular wall, lipid deposition thickening the intima, and stimulating the inflammatory response. This leads to injury to intima endothelial cells, increased permeability, fibrosis of media smooth muscle cells, increased arterial hardness, and decreased elasticity. The effect of electrolytes on hypertension is twofold. Lowering sodium intake and increasing potassium intake are known to be beneficial in reducing hypertension^43,44^, and adequate calcium intake is also advantageous for high blood pressure^45^. Most studies have demonstrated a protective effect of magnesium against hypertension^46^. The regulation of magnesium on blood pressure may include mechanisms such as acting as a calcium antagonist to regulate vascular tension and contraction, vascular endothelial function, aging and stiffness, vascular remodeling, oxidative stress, insulin resistance, inflammatory response, etc.

Hypertension is a prevalent clinical syndrome with multiple contributing factors. The occurrence of hypertension is attributed to various risk factors and the decompensation of blood pressure regulation mechanism. Simultaneously, hypertension and various risk factors can mutually influence and collectively contribute to the progression and aggravation of the disease. Hypertension frequently coexists with various conditions, including diabetes, hyperlipidemia, atherosclerosis, cardiovascular diseases, and cerebrovascular diseases. They may interact in a complex causal manner, exacerbating their respective pathological processes^47,48^.

In a preliminary study^49^, we demonstrated that the diameter of the thoracic aorta, particularly the middle descending aorta, significantly impacted masked hypertension and poorly controlled outcomes of hypertension. In this study, we additionally measured the cross-sectional area, volume, and centerline length of the thoracic aorta, in addition to the diameter of nine levels. Our study revealed significantly differences in all diameters, areas, volumes, and lengths of the ascending and descending aorta between non-hypertensive and hypertensive groups. Following dimensionality reduction and variable selection, the prediction model ultimately includes D3, D4, D6, D7, A1 and V7_8. The AUC for the training set and validation set is 0.735 and 0.767, respectively. In a previous study, the diameters and volumes of ascending, arching, and descending segments of the thoracic aorta were larger in hypertensive patients than in subjects with normal blood pressure (*P* < 0.001), and the differences persisted after adjusting for age^50^. According to Laplace's law, the size of the vascular lumen is inversely proportional to the thickness of the wall and directly proportional to the pressure^51^. To maintain the stability of circumferential pressure under the condition of constant vascular thickness, an increase in blood pressure inevitably leads to an expansion of diameter. Vasodilation mediated by blood flow occurs when a sudden increase in blood flow in the lumen induces shear force on the vascular wall, resulting in damage to vascular endothelial cells and subsequent vasodilation^25,52^.

We developed five distinct models based on conventional risk factors, biochemical markers, co-morbidity, and thoracic aorta imaging factors of the thoracic aorta measured on non-enhanced chest CT. All five models exhibited good diagnostic performance (AUC 0.735–0.836), along with robust calibration capabilities and significant clinical net benefits. Framingham Heart Study developed a short-term hypertension prediction model considering the interaction between age, gender, SBP, DBP, current smokers, parental hypertension, BMI, age and DBP^9^ under the premise of Caucasian patients without diabetes. However, it has been verified that the model’s extensibility is limited^10,11^. In reality, many chronic diseases tend to co-occur, such as diabetes and hypertension. Therefore, not excluding individuals with diabetes in the study is more in line with the actual clinical scenario. Numerous hypertension prediction models have been developed in Asia, including China, but they often incorporate only partial risk factors and location-specific variables. As an example, a prediction model for Kazakh herdsmen in Xinjiang not only included age, body mass index, blood lipid, and other factors but also considered dietary factors (such as yak butter often consumed in pastoral areas). The model achieved an AUC of 0.803 in modeling set and 0.809 in verification set^12^. Leveraging genetic and environmental factors, Li et al. built prediction models for systolic and diastolic blood pressure, yielding AUC values of 0.673 and 0.817, respectively^17^.

In summary, we developed and validated five hypertension prediction models using distinct predictors. Primary care workers can select from different prediction models, tailoring hypertension predictions for individual patients based on the available predictors. For example, when patients undergo chest CT for lung cancer screening, the AIMeasure model can predict the risk of hypertension; Conversely, when patients present additional clinical data like major biochemical laboratory examination and past medical history, the AITotalClinical model emerges as a valuable tool for predicting hypertension risk. This will enhance strategies for preventing and treating hypertension, effectively reducing and delaying the onset of adverse events of related to hypertension.

Nevertheless, certain limitations in this study warrant consideration. First of all, the study focused on patients undergoing routine health examinations in the general medical department of our hospital, with an age range spanning from 18 to 95 years old. Consequently, the predictive capacity of the model in different ethnic groups or specific populations remains uncertain. Secondly, the study exclusively incorporated risk factors accessible through routine examinations, excluding other predictors like economic status, educational level, psychosocial elements, and genetic markers. Lastly, as this study is a single-center cross-sectional study, the sample represents only a portion of the population in this region. Since hypertension risk factors can vary across different regions, the generalizability of this study is somewhat constrained, and the model's stability requires additional external validation.

## Conclusion

In this study, five hypertension risk prediction models were established based on clinical risk factors for hypertension and thoracic aorta image features measured on non-enhanced chest CT. These include the BasicClinical model, which relies on easily obtainable basic information; the TotalClinical model, which incorporates comprehensive clinical data such as basic information, biochemical indicators, and comorbidities; the AIMeasure model, focusing on thoracic aorta image features; the AIBasicClinical model, combining basic information and thoracic aorta image features; and the AITotalClinical model, integrating comprehensive clinical data with thoracic aorta image features.

Upon evaluation, all five models demonstrated favorable predictive and calibration capabilities, offering potential clinical utility. Consequently, in diverse clinical scenarios, patients can select the appropriate hypertension risk prediction model based on existing or accessible clinical or imaging data. This aids in the preliminary identification of high-risk hypertension patients and enhances the efficiency of primary prevention efforts. However, the generalizability of these models requires further assessment and external validation in large-scale studies in the future.

### Supplementary Information

Supplementary Information.

## Data Availability

The data presented in this study are available on reasonable request from the corresponding author.
