# Supplementary material for: Development of hypertension models for lung cancer screening cohorts using clinical and thoracic aorta imaging factors
Source: Sci Rep. 2024 Mar 22;14:6862. doi: 10.1038/s41598-024-57396-1 (PMC10957886; doi:10.1038/s41598-024-57396-1)
Supplement: Supplementary file 1 — Supplementary Information. [file 41598_2024_57396_MOESM1_ESM.docx]

| Features | Features | Features | Features |
| --- | --- | --- | --- |
| D1 | A1 | V0_1 | L1_2 |
| D2 | A2 | V1_2 | L2_3 |
| D3 | A3 | V2_3 | L3_4 |
| D4 | A4 | V3_4 | L1_4 |
| D5 | A5 | V1_4 | L4_5 |
| D6 | A6 | V4_5 | L5_6 |
| D7 | A7 | V5_6 | L4_6 |
| D8 | A8 | V4_6 | L6_7 |
| D9 | A9 | V6_7 | L7_8 |
| — | — | V7_8 | L8_9 |
| — | — | V8_9 | L6_9 |
| — | — | V6_9 | L1_9 |
| — | — | V0_9 | — |
| D: Diameter; A: Area; V: Volume; L: Length; The numbers 1-9 represent the 9 levels of thoracic aorta, as shown in supplementary Fig. S1; 0 indicates the starting point. And L0_1 was not calculated due to technical reasons. | | | |

Supplementary Table. S1 Description and interpretation of 43 features


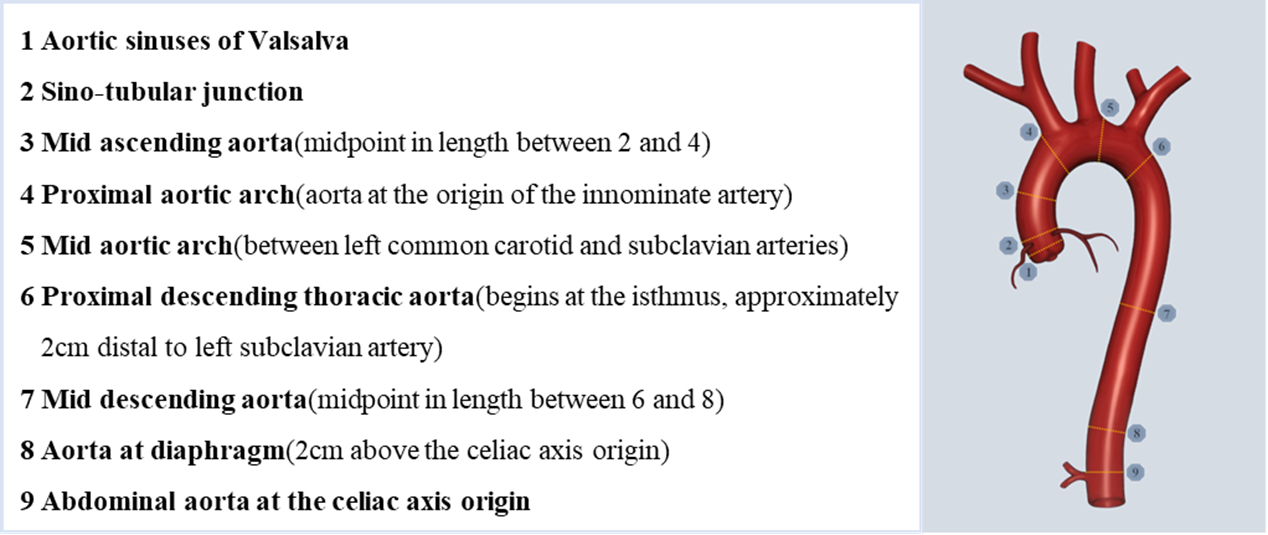


Supplementary Fig. S1 Schematic diagram of 9 levels of the thoracic aorta


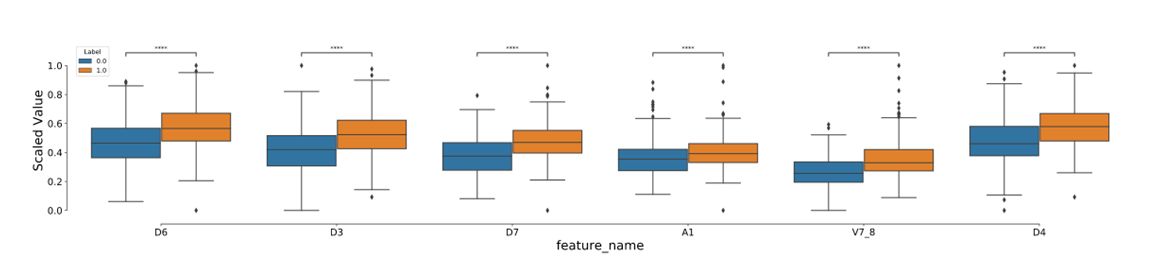
Supplementary Fig. S2 Differences in selected thoracic aorta features between the non-hypertension group (label=0, blue) and the hypertension group (label=1, orange)

The abbreviations correspond to those in Fig.3 and Table 1.

**
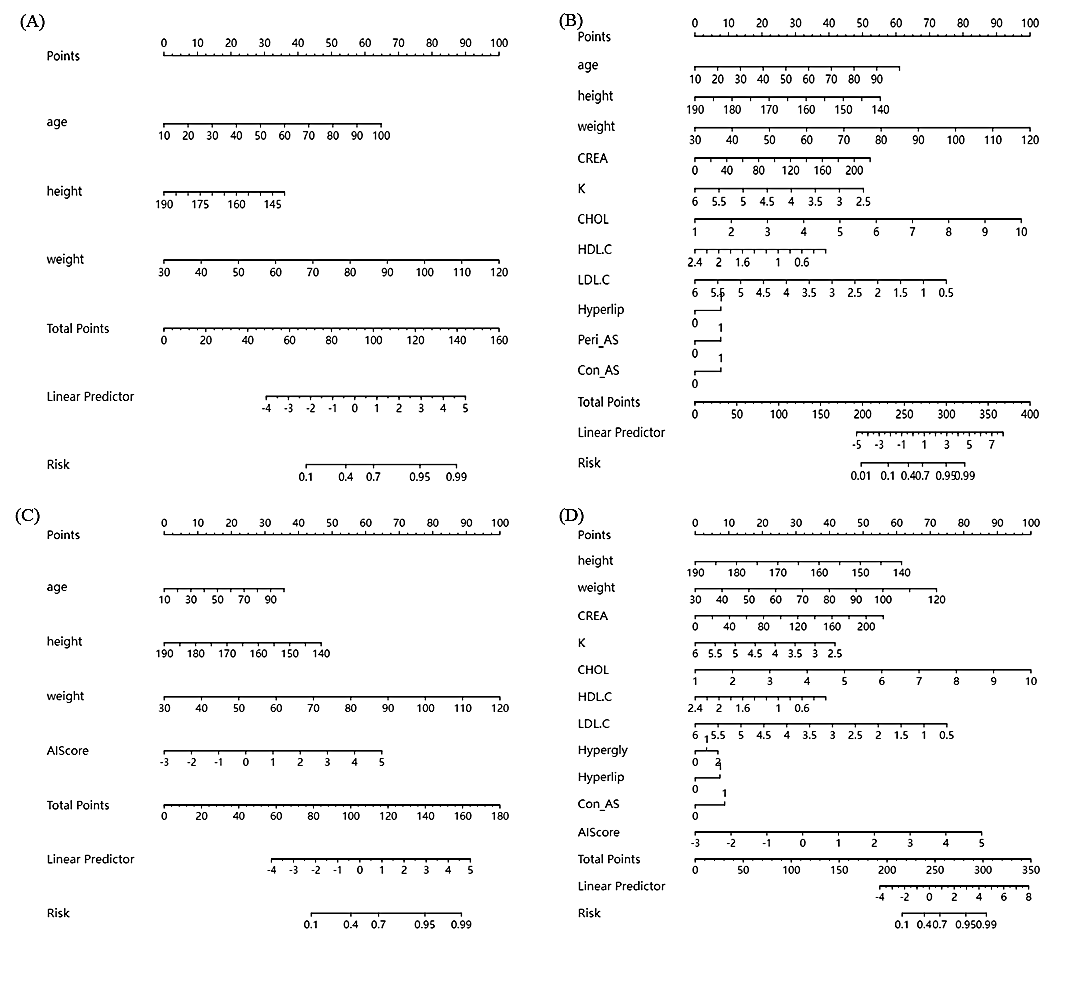
**

Supplementary Fig. S3 Nomogram of the four models for predicting the risk of hypertension

(A) BasicClinical model based on basic clinical data (including sex, age, height, weight, BMI, smoking history, drinking history, etc.); (B) TotalClinical model based on total clinical data (including the basic clinical data, serum biomarkers and concomitant diseases, etc.); (C) AIBasicClinical model combining the selected basic clinical factors and AI-score; (D) AITotalClinical model incorporating the selected valuable clinical factors and AI-score.

The abbreviations are the same as in Table 1.
